# Impact of Continuous Estroprogestin Treatment on Circulating Microparticle Levels in Deep Endometriosis Patients

**DOI:** 10.3390/ijms241411802

**Published:** 2023-07-22

**Authors:** Pilar Carrillo Torres, María Ángeles Martínez-Zamora, Dolors Tàssies, Helena Castillo, Meritxell Gracia, Georgina Feixas, Joan Carles Reverter, Francisco Carmona

**Affiliations:** 1 Gynaecology Department, Clinic Institute of Gynaecology, Obstetrics and Neonatology (ICGON), Hospital Clinic of Barcelona, Universitat de Barcelona, 08007 Barcelona, Spain; 2 Hemotherapy and Hemostasis Department, Clinic Institute of Hemato-Oncological Disease (ICMHO), Hospital Clínic of Barcelona, Universitat de Barcelona, 08007 Barcelona, Spain

**Keywords:** endometriosis, hormonal therapy, combined oral contraceptives, estroprogestin treatment, coagulation, circulating microparticles, tissue factor

## Abstract

There has been increasing interest in the study of new pathogenic mechanisms in endometriosis (END), including the coagulation/fibrinolysis system and its link with inflammation and tissue remodeling. It has been suggested that END patients, especially with deep-infiltrating (DE) forms, could present a hypercoagulable state revealing higher levels of proinflammatory and procoagulant markers, such as total circulating microparticles (cMPs) and cMP-TF (tissue factor), released by cells in response to damage, activation, or apoptosis. However, no previous study has assessed the effect of END hormonal treatments on cMP and cMP-TF levels. Therefore, the aim of this study was to evaluate the impact of these treatments on cMP and cMP-TF levels in DE patients. Three groups were compared: DE patients receiving a continuous combined oral contraceptive regimen (CCOCR) (n = 41), DE patients without CCOCR (n = 45), and a control group (n = 43). cMP and cMP-TF levels were evaluated in platelet-free plasma. A significant decrease in the total cMP levels was found in the DE group with CCOCR versus the group without CCOCR, reflecting a higher chronic inflammatory status in DE patients that decreased with the treatment. cMP-TF levels were higher in DE patients receiving CCOCR versus those not receiving CCOCR, suggesting that treatments containing estrogens play a predominant role in suppressing the inhibitory pathway of TF.

## 1. Introduction

Endometriosis (END) is a hormone-dependent disease caused by the presence of endometrial-like tissue outside the uterine cavity, inducing a systemic chronic inflammatory reaction [1,2]. It has a prevalence of 10% in women of reproductive age, and despite its high prevalence, a significant delay in diagnosis has been reported, with a gap from first symptoms to treatment ranging from 8 to 12 years [1,2]. END may present itself with several symptoms such as chronic pelvic pain, dysmenorrhea, dyspareunia, dysuria, and/or dysquezia, although, in other cases, the main symptom may be infertility [1,2]. However, one major point that most of the patients will agree on is a significant impairment in their global quality of life, an important matter that has gained interest in the past few years [3,4].

Three well-recognized phenotypes have been described: superficial peritoneal endometriosis (SP), ovarian endometriomas (OE), and deep-infiltrating endometriosis (DE), the latter being the most severe form of the disease [5]. There is general agreement that END is associated with a local inflammatory response and that for endometriotic lesions to appear and persist, other complementary phenomena, aside from the classical theories describing their origin, are needed [6].

In the past years, there has been a growing interest in the study of new pathogenic mechanisms in END that include the coagulation/fibrinolysis system and its link with inflammation and tissue remodeling [7,8] and that suggest that patients with END could present a hypercoagulable state [9,10]. Therefore, there has been notable interest in the study of new hemostasis markers, emphasizing novel perspectives that could revolutionize the way we understand END [11,12,13,14].

Among the new markers studied are the circulating microparticles (cMP) and the cMP-TF (a subset of cMP that contains tissue factor (TF)) [15]. cMP are membrane-bound vesicles < 1 μm in diameter that are formed by the release of membrane fragments from multiple cells, such as platelets or endothelial cells, in response to damage, activation, or apoptosis [16]. cMPs have been identified in human plasma, urine, saliva, and cerebrospinal fluid and may act as markers of key functions in hemostasis, immunity, inflammation, and angiogenesis [17,18]. Higher cMP levels have been found in conditions in which vascular dysfunction and inflammation are important pathophysiological mechanisms, such as cardiovascular disease, diabetes, preeclampsia, thrombotic disorders, and DE [19,20]. Moreover, several studies have shown that treatment for these pathologies may lower cMP levels [21,22], and these levels may be surrogate markers for therapy responsiveness [23]. cMP exert their procoagulant function by expressing phosphatidylserine (PS) on their surface, which is needed for coagulation factors’ anchorage and activation [16]. A subgroup of cMP can, additionally, express TF. TF, also known as coagulation factor III, initiates the activation of the coagulation cascade by forming a complex with coagulation factor VIIa [24]. Furthermore, TF is known to have a proangiogenic function as well as a proinflammatory role in sepsis or thrombotic scenarios [25]. In END patients, an elevation of TF expression has been reported in the glandular epithelial cells of eutopic and ectopic endometrium [26]. However, this local increase of TF may not be reflected in cMP levels containing TF, with similar levels of cMP-TF in END patients and controls [15].

Current therapeutic options for END are based on long-term hormonal treatments (HT), with surgical treatment only indicated in specific cases [27]. Continuous combined oral contraceptive regimens (CCOCR) (estroprogestins) and progestins are currently considered first-line treatments and have been demonstrated to improve clinical symptoms and radiological lesions [28,29]. Nevertheless, there is limited scientific evidence about their biological effects on END patients. Estroprogestins are known to have an impact on coagulation [30,31]; however, to our knowledge, this issue has not been evaluated in patients with END receiving HT. Based on this scenario, we aimed to evaluate the impact of continuous estroprogestins on cMP and cMP-TF levels in DE patients.

## 2. Results

### 2.1. Patient Characteristics

**Figure 1:**
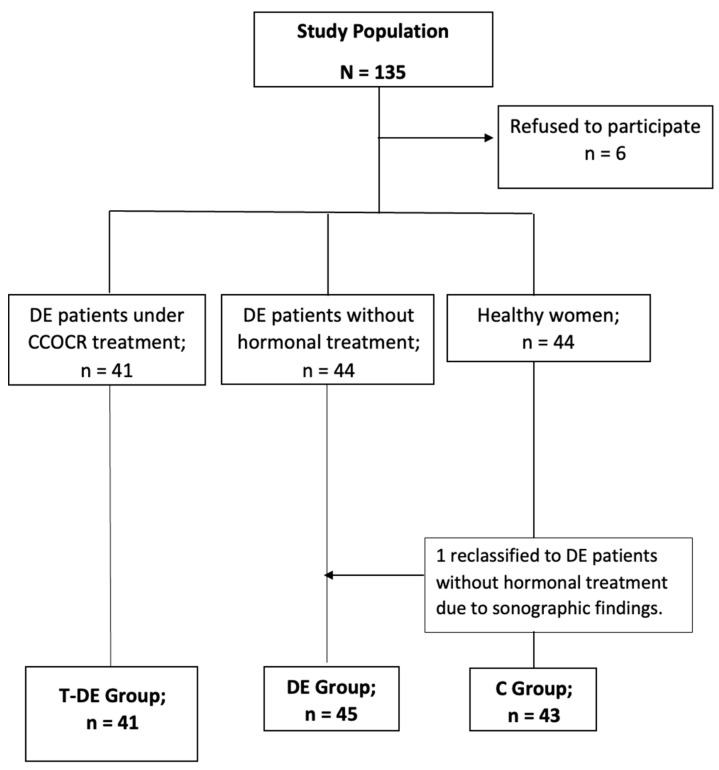
Patient flow chart of inclusion and drop-out of the patients included in the study. DE: deep endometriosis, CCOCR: continuous combined oral contraceptive regimen, C: control.

A total of 129 patients were finally evaluated: 41 were patients diagnosed with DE under treatment with CCOCR (T-DE Group), 45 were DE patients without HT (DE Group), and the remaining 43 acted as the control group (Figure 1). The baseline characteristics of the patients are shown in Table 1. The types and doses of estroprogestins administered to the T-DE group are described in Table 1. No serious adverse events were reported during the study period in patients receiving HT.

T-DE Group: women diagnosed with deep endometriosis receiving hormonal treatment with a continuous combined oral contraceptive regimen (CCOCR); DE Group: women diagnosed with deep endometriosis not receiving any type of hormonal treatment; and C Group, which acted as a control group and was formed by women without endometriosis and who were not receiving hormonal treatment for other reasons. Results are expressed as numbers and percentages or mean ± standard deviation. N/A: not applicable. CCOCR: continuous combined oral contraceptive regimen; DE: deep endometriosis; EE: etinylestradiol; LNG: levonorgestrel; NA: not applicable; BMI: body mass index.

### 2.2. Evaluation of Total cMP Levels 

**Figure 2:**
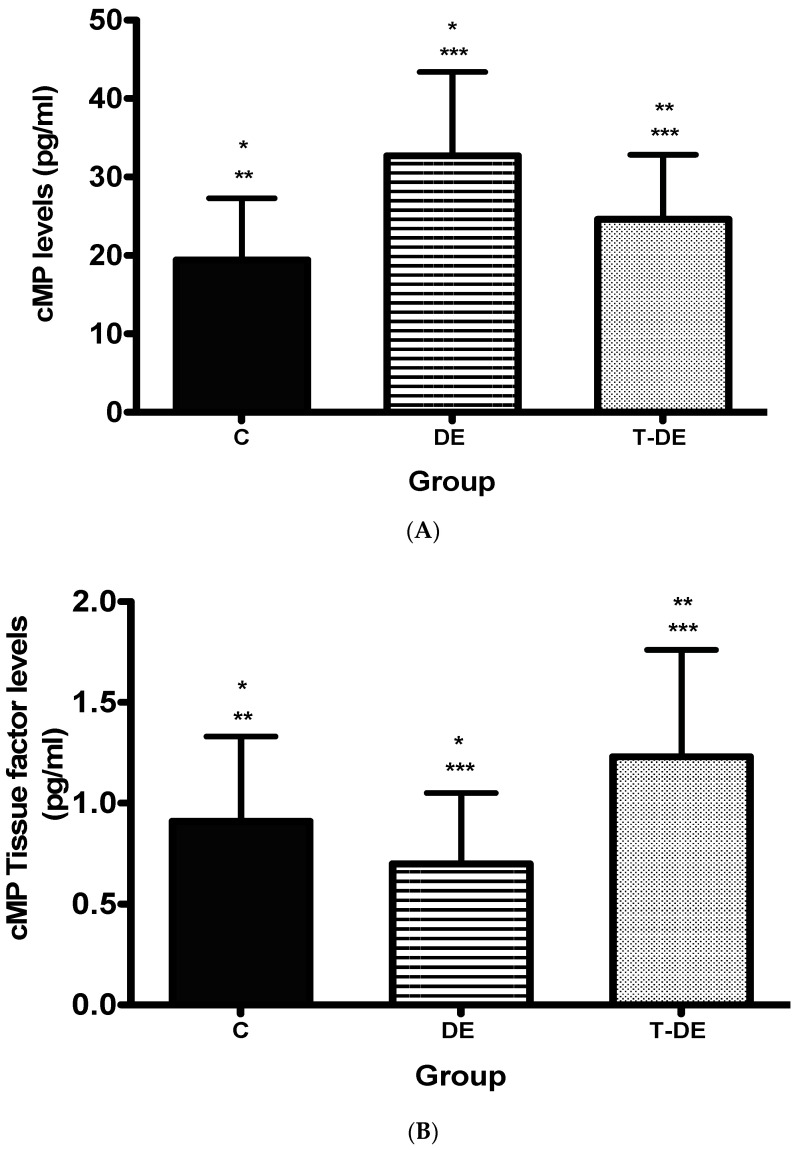
(**A**) Total circulating microparticle levels in the three groups. (**B**) Microparticle tissue factor levels in the three groups. cMP: circulating microparticles; T-DE Group: women diagnosed with deep endometriosis receiving hormonal treatment with a continuous combined oral contraceptive regimen (CCOCR); DE Group: women diagnosed with deep endometriosis who were not receiving any type of hormonal treatment; and C Group acted as a control group and was formed by women without endometriosis who were not receiving hormonal treatment for other reasons. Results are expressed as the mean minus the standard deviation. Superscripts show statistical differences: (**A**) *p* < 0.001 *, *p* < 0.031 **, *p* < 0.005 ***. (**B**) *p* = 0.066 *, *p* = 0.154 **, *p* < 0.002 ***.

The total cMP levels were higher in the DE group compared to the C Group (32.71+/−10.67 vs. 19.43+/−7.85). Patients in the T-DE Group showed statistically significantly lower cMP levels (24.61+/−8.24) than the DE group but higher than the C Group (Figure 2A).

### 2.3. Evaluation of cMP-TF Levels

Regarding cMP-TF levels, there were no differences between the DE group (0.70+/−0.35) and the C group (0.91+/−0.42). Higher cMP-TF levels were found in the T-DE group (1.23+/−0.53) compared to the DE group. No statistical differences were found in cMP-TF levels when comparing the T-DE and the C groups (Figure 2B).

## 3. Discussion 

In the last few years, research regarding END has focused on understanding the new pathogenic mechanisms of this entity. Among the phenomena hypothesized, several studies have found that END could present a state of hypercoagulability or hypofibrinolysis leading to an increase in cMP, which could play a role in its pathogenesis [9,10,15]. We found significantly higher cMP levels in DE patients compared to controls and a significant decrease in cMP levels in DE patients with CCOCR treatment. Additionally, increased cMP-TF levels were found in DE patients with CCOCR treatment compared to DE patients without HT.

The finding of higher levels of total cMP in END patients is in consonance with publications that reported increased cMP levels in inflammatory diseases such as autoimmune disorders, cardiovascular diseases, and infectious diseases [15,16,19,22,23,24,25,26,27,28,29,30,31,32]. Our group recently reported a prospective case-control study comparing 65 patients with surgically confirmed END (37 with DE lesions) and 33 women without surgical findings of END. Total cMP plasma levels were found to be higher in the END group compared with the control group (*p* < 0.0001). The subanalysis of END patients with DE or without DE showed that total cMP levels were higher in the DE group (*p* < 0.001) [15]. 

Regarding the effect of treatments on cMP levels, it is known that in entities, such as malignant hematological disease or diabetes [22,23], cMP levels decrease when the disease is effectively treated. We found a significant decrease in cMP levels in END treated with HT that could be explained by the fact that HT can suppress cyclical bleeding in endometriotic lesions, and subsequently, the activation of platelets, which leads to the formation of cMP and inflammation [33,34]. 

It is classically known that HT may also provide a hypercoagulability status mainly related to oral estrogen administration, as transdermal estrogen and transdermal or oral progesterone are not associated with a higher risk of thrombosis [35]. The reason for the increased risk can be partly explained by a procoagulant status due to changes in the coagulation balance with decreased levels of inhibitory coagulation factors [36]. Among these, the reduction in tissue factor pathway inhibitor (TFPI) is probably the most important mechanism, and, together with decreased levels of protein S, predicts activation of coagulation and acquired resistance to activated protein C [36]. Therefore, the higher cMP-TF levels in the DE group receiving CCOCR found in our study could be explained by the role of estrogens in suppressing the TFPI and subsequently increasing cMP-TF levels [37]. 

Other studies have reported an increased local expression of TF in eutopic and ectopic endometrium [26,38,39] in END patients without HT. This local expression could not be reflected in plasma levels, according to the results of a previous study by our group that found no differences in cMP-TF levels in END patients compared to healthy patients [15]. 

Then, although HT can increase cMP-TF levels, these changes do not seem to be large enough to influence the risk of thrombosis, and in END patients, the benefit of HT outweighs the potential side effects, as suggested by the global decrease of total procoagulant activity found in our study.

The strengths of this study include strict inclusion and exclusion criteria and the evaluation of a homogeneous sample of DE patients. It is noteworthy that patients with concomitant adenomyosis were excluded to avoid bias. The frequent association between adenomyosis and END is well-known, and adenomyosis may have influenced the laboratory results.

Several limitations of our study should be considered for data interpretation. Firstly, we enrolled a relatively small number of patients, although the number was comparable to other studies published in the literature [9,11,14,15,20,21,33]. Secondly, only treatment with CCOCR was explored without comparing this treatment with other frequently used HT, such as progestogens. Finally, the impact of CCOCR was not evaluated in a control group under HT, which would have been interesting to compare with the DE group and further elucidate the impact of HT on DE.

Based on this scenario, future research should consider the performance of a larger study investigating cMP and cMP-TF as well as other inflammation and coagulation markers focusing on the fibrinolytic system and exploring their impact on other HT and different doses and their possible use as biomarkers for decision-making algorithms concerning HT.

## 4. Materials and Methods

### 4.1. Study Design

A single-center longitudinal prospective observational case-control study was conducted at the Department of Gynecology of the Hospital Clinic of Barcelona, a tertiary university hospital in Spain, and a referral center for the diagnosis and treatment of END. The study was approved by the local Ethical Committee according to prevailing regulations. Written informed consent was obtained from all participants.

### 4.2. Participants

A total of 135 consecutive patients were recruited and were divided into 3 study groups to assess the previously stated objectives. To be eligible, the patients had to be >18 years old, premenopausal women who had undergone transvaginal sonography (TVS) in our center and presented the following characteristics: the T-DE Group were women diagnosed with DE receiving HT with a CCOCR; the DE Group were women diagnosed with DE who were not receiving any type of HT; and the C Group was a control group made up of women without END and who were not receiving HT for other reasons.

The exclusion criteria were a history of past or present malignancy, endocrine, cardiovascular and systemic diseases, pregnancy or breastfeeding ≤ 6 months before sample collection, premature ovarian failure or menopausal status, diagnosis of endometrial hyperplasia or polyps, uterine leiomyomata, adenomyosis, or having had an inflammatory disease or infectious condition ≤6 months before sample collection. Patients with surgical criteria or in whom TVS was not possible (e.g., virgin patients) were excluded. The use of gonadotrophin-releasing hormone analogs in the past 6 months or the use of other HT in the past 3 months in the 3 groups were also exclusion criteria. Figure 1 shows the flow chart of inclusion and drop-out of the patients included in the study.

Patients in the DE Group did not receive HT mainly due to a recent diagnosis (n = 39) or less frequently because of pregnancy desire (n = 6).

### 4.3. Study Procedures

Venous blood samples were collected at recruitment. The samples were obtained in tubes containing 3.8% trisodium citrate (1:9, *v*:*v*) (Becton Dickinson, Rutherford, NJ, USA), and platelet-free plasma was immediately obtained by double centrifugation: first at 2000× *g* for 10 min at 22 °C, and then at 5000× *g* for 10 min at 4 °C. Plasma was aliquoted and stored at −80 °C.

The total procoagulant activity of cMPs was tested in plasma using a commercial functional assay (Hyphen BioMed, Neuville, France Catalog number: 521096) based on the property of annexin-V, immobilized onto plastic wells to bind PS expressed in cMPs, as previously described [15]. In brief, plasma samples were placed onto plate microwells coated with streptavidin and biotinylated annexin V. After incubation and washing, bovine factor Xa–Va and human prothrombin were added. After further incubation, a thrombin-specific chromogenic substrate was added. The reaction was stopped with 2% citric acid after 10 min, and absorbance was measured at 405 nm. Calibrators with known amounts of PS were used to obtain a standard curve. The results were expressed as nanomolar PS equivalent (nM PS eq) [15]. The detection limit of the assay was 0.05 nM, the intra-assay coefficient of variation (CV) was 5% and the interassay CV was 8%.

cMP-TF activity in plasma was measured using a commercial ELISA kit (Zymuphen MP-TF, Hyphen Biomed, Neuville, France) as previously described in a study by our research group [15]. In brief, the samples are first introduced into the wells of the microplate coated with a murine monoclonal antibody specific for the human TF extracellular domain, which does not interfere with TF activity. cMP-TF present in the sample bind to the solid phase through an epitope localized in the extracellular domain of TF. Following overnight incubation and a washing step, the wash solution is immediately introduced into the wells. Then, Factor VIIa and Factor X are added. The TF–FVIIa complex forms and subsequently activates Factor X into activated Factor X (FXa) on the surface of the anionic phospholipids present in the cMP and in the presence of Ca2+. After that, a specific substrate for FXa is added and reacts with FXa producing a yellow color. The absorbance is recorded at 405 nm on a spectrophotometer and is directly proportional to the amount of cMP-TF present in the sample. A calibration curve is constructed. The calibration is validated when the quality controls are measured within their acceptance range, indicated for each lot in the instructions provided in the kit. The results are expressed as pg/mL. The detection threshold is ≤1 pg/mL. For cMP-TF, the intra-assay CV was 7% and the inter-assay CV was 10%

All patients underwent high-resolution 2D-3D TVS using an endovaginal probe (type RIC5-9, Voluson V730 Expert, GE Healthcare, Milwaukee, WI, USA) and the diagnosis of END was established following the International Deep Endometriosis Analysis (IDEA) group consensus [40].

### 4.4. Statistical Analysis

The sample size was decided arbitrarily, based on previous studies analyzing cMP/cMP-TF in other inflammatory diseases and biomarkers in END [9,11,14,15,20,21,33]. Categorical variables are expressed as count and percentages, and continuous variables as mean and standard deviation. The distribution of categorical variables was compared with the Chi-square test, and quantitative variables with the ANOVA test using the post hoc Bonferroni multiple comparison test, when appropriate. Statistical significance was set at *p* < 0.05. Statistical analysis was performed with the Statistical Package for the Social Sciences software, release 25.0 for Windows (SPSS, Chicago, IL, USA).

## 5. Conclusions

The results of our study provide data showing higher levels of total cMP in DE patients, reflecting a higher inflammatory and/or procoagulant systemic status, and that CCOCR decreases cMP levels, reflecting a systemic decrease in inflammation. cMP-TF levels were increased in the CCOCR group and may reflect the role of estrogens in suppressing the inhibitory pathway of TF and thereby increasing cMP-TF levels. Nonetheless, further research is warranted to confirm our findings and assess the exact role of cMP and cMP-TF in the pathophysiological mechanisms of END and the effects of HT.

## Figures and Tables

**Table 1:** Baseline clinical and demographic data of the three study groups.

	T-DE Group (n = 41)	DE Group (n = 45)	C Group (n = 43)	*p* Value
Age (years)	32.5+/−5.7	33.2+/−5.3	33.8+/−4.9	0.5
BMI (Kg/m^2^)	23.3+/−3.6	22.9+/−3.2	23.1+/−2.9	0.5
Tobacco use	6 (14.6)	8 (17.8)	9 (20.9)	0.6
Previous pregnancy	19 (46.3)	22 (48.9)	20 (46.5)	0.3
**DE forms**				
Uterosacral ligaments/torus uterinus	37 (90.2)	41 (91.1)	N/A	0.2
Recto-sigmoid	8 (19.5)	9 (20.0)	N/A	0.5
Vesical	2 (4.9)	1 (2.2)	N/A	0.9
Ureteral	0 (0.0)	1 (2.2)	N/A	1
Vaginal	1 (2.4)	0 (0.0)	N/A	1
**Ovarian endometriomas**				
Unilateral	17 (41.5)	18 (40.0)	N/A	0.7
Bilateral	2 (4.9)	3 (6.7)	N/A	0.1
**Duration of CCOCR (months)**	38+/−8.5	N/A	N/A	N/A
**Type of CCOCR**				
EE 20 ug + drospirenone 3 mg	11 (26.8)	N/A	N/A	N/A
EE 30 ug + drospirenone 3 mg	4 (9.8)	N/A	N/A	N/A
EE 30 ug + dienogest 2 mg	19 (46.3)	N/A	N/A	N/A
EE 20 ug + LNG 100 ug	7 (17.1)	N/A	N/A	N/A

## Data Availability

The data presented in this study are available on request from the corresponding author.
